# Perceptions of Menthol Cigarettes Among Twitter Users: Content and Sentiment Analysis

**DOI:** 10.2196/jmir.5694

**Published:** 2017-02-27

**Authors:** Shyanika W Rose, Catherine L Jo, Steven Binns, Melissa Buenger, Sherry Emery, Kurt M Ribisl

**Affiliations:** ^1^ The Schroeder Institute for Tobacco Research and Policy Studies at Truth Initiative Washington, DC United States; ^2^ Department of Health Behavior Gillings School of Global Public Health University of North Carolina at Chapel Hill Chapel Hill, NC United States; ^3^ Health Media Collaboratory NORC at the University of Chicago Chicago, IL United States; ^4^ Lineberger Comprehensive Cancer Center University of North Carolina at Chapel Hill Chapel Hill, NC United States

**Keywords:** tobacco products, menthol, smoking, social media, Twitter messaging, policy, public opinion

## Abstract

**Background:**

Menthol cigarettes are used disproportionately by African American, female, and adolescent smokers. Twitter is also used disproportionately by minority and younger populations, providing a unique window into conversations reflecting social norms, behavioral intentions, and sentiment toward menthol cigarettes.

**Objective:**

Our purpose was to identify the content and frequency of conversations about menthol cigarettes, including themes, populations, user smoking status, other tobacco or substances, tweet characteristics, and sentiment. We also examined differences in menthol cigarette sentiment by prevalent categories, which allowed us to assess potential perceptions, misperceptions, and social norms about menthol cigarettes on Twitter. This approach can inform communication about these products, particularly to subgroups who are at risk for menthol cigarette use.

**Methods:**

Through a combination of human and machine classification, we identified 94,627 menthol cigarette-relevant tweets from February 1, 2012 to January 31, 2013 (1 year) from over 47 million tobacco-related messages gathered prospectively from the Twitter Firehose of all public tweets and metadata. Then, 4 human coders evaluated a random sample of 7000 tweets for categories, including sentiment toward menthol cigarettes.

**Results:**

We found that 47.98% (3194/6657) of tweets expressed positive sentiment, while 40.26% (2680/6657) were negative toward menthol cigarettes. The majority of tweets by likely smokers (2653/4038, 65.70%) expressed positive sentiment, while 91.2% (320/351) of nonsmokers and 71.7% (91/127) of former smokers indicated negative views. Positive views toward menthol cigarettes were predominant in tweets that discussed addiction or craving, marijuana, smoking, taste or sensation, song lyrics, and tobacco industry or marketing or tweets that were commercial in nature. Negative views toward menthol were more common in tweets about smoking cessation, health, African Americans, women, and children and adolescents—largely due to expression of negative stereotypes associated with these groups’ use of menthol cigarettes.

**Conclusions:**

Examinations of public opinions toward menthol cigarettes through social media can help to inform the framing of public communication about menthol cigarettes, particularly in light of potential regulation by the European Union, US Food and Drug Administration, other jurisdictions, and localities.

## Introduction

Menthol is a characterizing flavor that imparts a minty flavor and a cool sensation masking the harshness of cigarette smoke [1,2]. Menthol cigarettes comprised 31% of market share for cigarettes sold in the United States in 2012 [3]. Use of menthol versus nonmenthol cigarettes is higher among African American, female, younger, and lesbian, gay, bisexual, or transgender (LGBT) smokers [4-8]. For instance, among smokers, 84% of African Americans versus 24% of whites smoke menthol cigarettes [6], and adolescent (57%) and young adults (45%) use menthol cigarettes at higher rates than all ages of older smokers [8]. All of these groups have been targeted by tobacco industry menthol cigarette marketing [9,10].

The US Food and Drug Administration (FDA) banned characterizing flavors in cigarettes except for menthol or tobacco flavor in 2009 [11]. Menthol was exempted pending further review. In 2011, the FDA’s Tobacco Products Science Advisory Committee (TPSAC) issued a report concluding that “Removal of menthol cigarettes would benefit public health in the United States” [12]. TPSAC and a subsequent FDA report [13] concluded that menthol cigarette use is likely associated with increased smoking initiation, increased levels of nicotine addiction, and reduced smoking cessation success, particularly among African Americans. Moreover, menthol cigarettes present the same disease risk as nonmenthol cigarettes [14], with smoking causing up to two-thirds of deaths in smokers [15]. The FDA has not yet decided on whether to ban menthol cigarettes [16]. However, other jurisdictions are moving forward with menthol cigarette bans and restrictions. Brazil became the first country to pass legislation banning menthol and flavors from tobacco products in 2012, but the ban has not yet taken effect [17]. In 2014, the city of Chicago enacted a ban on menthol and flavored tobacco product sales near schools. The European Union (EU) also banned menthol cigarettes with a 2020 implementation date [17]. In 2015, some Canadian provinces prohibited flavored and menthol cigarettes and tobacco sales [17].

Understanding perceptions of menthol cigarettes given this changing global regulatory environment is critical to understanding how to best communicate to the general public about these products prior to widespread policy implementation. Perceptions of menthol cigarettes among both smokers and the general public may contribute to use [9,18-21]. A study of US adolescents and young adults found that endorsing promenthol cigarette beliefs (positive sentiment) was associated with intention to use menthol cigarettes among noncurrent menthol cigarette users and intention to use other tobacco products among current menthol cigarette smokers not currently using other products [22].

Commonly reported positive perceptions of menthol cigarettes are that they are more cooling or refreshing than nonmenthol cigarettes [18,22], have a medicinal effect when the smoker is sick [18,23], and are part of African American culture or tradition [24]. Beliefs that menthol cigarettes are less harmful than nonmenthol cigarettes appear more common among African American smokers [18]. In negative sentiments, among current smokers, 22% believe that menthol cigarettes are more addictive, while 12.1% believe that menthol cigarettes are harder to quit smoking than nonmenthol cigarettes [21].

Most studies of perceptions of menthol cigarettes have been conducted through surveys or focus groups [18,21,23-26]. Analyzing the amount and content of social media messages is a relatively new and promising way to gather information about health-related topics [27,28]. On Twitter, users can share 140-character messages called *tweets* with their followers and the general public. The forum is popular, with the number of daily tweets topping 500 million [29]. An estimated 23% of the US adult online population used Twitter in 2014 [30]. Twitter also reaches a global audience with an estimated 316 million active users worldwide [31].

Used disproportionately by younger and racial and ethnic minority populations who also disproportionately use menthol cigarettes [30], Twitter may provide insight into use and perceptions of these products. Several studies have examined Twitter conversations about electronic cigarettes [32-36] and hookah [34,37]. However, to our knowledge, no study has examined Twitter conversations about menthol cigarettes. As in prior Twitter studies of tobacco, we used a content analysis approach [35-37] and coded specifically for sentiment [35,38] toward menthol cigarettes. Our purpose was to identify the content and frequency of conversations about menthol cigarettes, including themes, populations, user smoking status, other tobacco or substances, tweet characteristics (eg, song lyrics or pop culture references, commercial tweets), and sentiment. We also examined differences in menthol cigarette sentiment by prevalent categories, which allowed us to assess perceptions and potential misperceptions and social norms about menthol cigarettes on Twitter. This approach can inform communication about these products, particularly to subgroups who are at risk for menthol cigarette use.

## Methods

### Design

**Figure 1:**
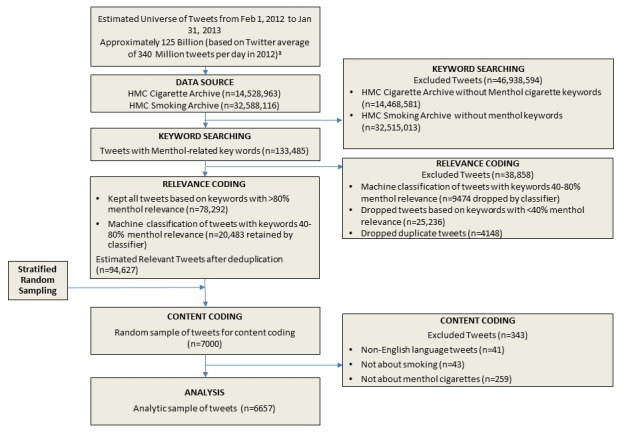
Overall study design and process for collecting menthol-relevant tweets for coding and analysis.^a^Data source: Internet Live Stats. HMC: Health Media Collaboratory.

We conducted a search to define a longitudinal set of tweets related to menthol cigarettes between February 1, 2012 and January 31, 2013 (shown in Figure 1 [39]). This time period provides a year of data and covers a time period prior to regulatory action on menthol cigarettes globally.

### Data Source

This observational study used data collected by the Health Media Collaboratory (HMC) [40]. The HMC accessed Twitter status updates through the Gnip PowerTrack Firehose (Gnip, Inc) [41], which prospectively collects all Tweets from public Twitter feeds.

The HMC created two archives that collected all Twitter status updates containing the keywords “cig” or “cigarette” (known as the cigarette archive) or the keyword “smoking” (the smoking archive). We chose those keywords after an extensive process of precision testing of terms likely to obtain tobacco-related content (Health Media Collaboratory, *Procedures Manual*, unpublished, 2012). The Gnip PowerTrack collected the content of the tweet, metadata such as username (who made the tweet), date and time the update was made, and hashtags (user-generated descriptive tags).

### Keyword Searching

At the time of our search for menthol cigarettes, the smoking archive had 32,588,116 tweets and the cigarette archive had 14,528,963. We identified an initial set of keywords including general terms, major menthol cigarette brands, slang terms, and common misspellings that we expected to generate relevant menthol cigarette-relevant content (see Table 1). We filtered the smoking and cigarette archives for each term, resulting in 133,485 tweets.

**Table 1:** Keywords used to identify menthol cigarette-related tweets and assessment of relevance.

Keywords		Cigarette archive		Smoking archive	
		Number of tweets found	% of relevant tweets in 100-tweet sample or all if <100 with keyword	Number of tweets found	% of relevant tweets in 100-tweet sample or all if <100 with keyword
**General terms**					
	Menthol	22435^a^	52^a^	6897	93
	Mentholated	161	96	47	96 (n=45)
	Menthols	1388	100	2956	100
**Brands**					
	Camel Crush	1254	100	590	100
	Kool	3061^b^	35^b^	7966^b^	17^b^
	Kools	345	100	1101	100
	Newport	12553	88	22645	98
	Newports	5985	100	15709	98
	Salem	4078^b^	20^b^	803^b^	12^b^
	Salems	33^b^	100 (n=33)	94	91 (n=86)
	Skyline (Marlboro)	174^b^	28^b^	617^b^	5^b^
**Misspelling**					
	Methol	98	96 (n=94)	61	95 (n=58)
	Methols	23	96 (n=22)	26	100 (n=26)
**Slang**					
	Newp	125	100	322	98
	Newps	132	100	307	100
	Port	6972^b^	1^b^	7522^a^	77^a^
	Ports	1565^b^	26^b^	5440	100

^a^Tweets were subject to machine learning assessment.

^b^Tweets were dropped as having <40% relevant content. All other tweets were retained, as keyword precision tests were ≥80% menthol cigarette related based on relevance coding.

### Relevance Coding

Once the searches were completed, 2 coders independently coded 100 randomly selected tweets from each keyword or all tweets if less than 100 for menthol relevance. We calculated a kappa statistic for each term to assess interrater reliability. For any term with at least .8 kappa score, one author (SWR) verified the coding and generated a measure of precision (percentage of identified tweets relevant to menthol cigarettes). For any keyword less than .8 kappa score, we coded another 100 tweets until we reached this level of interrater reliability. Of the 42 keywords searched, we achieved a kappa of at least .8 on the first attempt in 41 (98%) keywords.

We used DiscoverText (Texifter, LLC), a cloud-based text analytic software with active machine learning algorithms, allowing human coders to “train” the computer on a coding schema using a selection of tweets. If the keyword generated less than 40% of relevant tweets from the 100-tweet sample, we dropped all tweets with that keyword. If a keyword generated 80% or more relevant tweets in the 100-tweet sample, we kept all tweets. If the keyword generated 40% to 80% relevant tweets, we used the machine learning to assign a probability of a given tweet being menthol cigarette relevant, validated the machine estimates using a second 100-tweet sample coded by human coders, and retained only tweets with greater than 0.8 probability of being menthol cigarette relevant based on the machine classification. Of the 29,957 tweets assessed by the classifier, we retained 68.37% (n=20,483).

After merging and deduplicating these sets of tweets across archives and keywords, we ended with 94,627 unique tweets to include in content coding.

### Content Coding

We developed a codebook (see Table 2 for brief description of codes; Multimedia Appendix 1 provides the broader codebook) for directed content analysis [42], building on empirically derived constructs but allowing for emergent categories in the following areas: themes, populations, other tobacco or substances, tweet characteristics, user smoking status, and sentiment toward menthol cigarettes.

**Table 2:** Brief description of codes.

Categories		Definitions
**Themes (topical content dealing with perceptions of menthol cigarettes)**		
	Taste/sensation	Taste, smell, or sensation (eg, cooling, minty, refreshing) of menthol cigarettes.
	Health	Perceived health benefits or harms of menthol cigarettes, or beliefs about medicinal effects of menthol cigarette use.
	Cessation	Desire or lack of desire to quit smoking menthol cigarettes; beliefs about whether menthol cigarettes are harder or easier to quit than nonmenthol cigarettes.
	Addiction	Addiction, cravings, or desire for their lack of menthol cigarettes, beliefs that menthol cigarettes are more or less addictive than nonmenthol cigarettes.
	Smoking behavior	Act or process of smoking menthol cigarettes, including time or place of smoking.
	Tobacco control policies	Tobacco control policies or the impact of tobacco control policies.
	Industry/marketing	Advertising, promotion, labeling, or packaging of menthol cigarettes, including references to tobacco companies and protobacco organizations.
**User smoking status (likely smoking status of the Twitter user)**		
	Smoker	User writing tweet is likely to be a smoker.
	Former smoker	User writing tweet is likely to be a former smoker.
	Nonsmoker	User writing tweet is likely to be a nonsmoker. Also use this code for tweets from antitobacco organizations.
	Unknown	Cannot determine smoking status of user writing tweet.
**Tweet characteristics (additional information about tweets)**		
	Commercial	Branded promotional messages; URLs linking to commercial websites; usernames indicating affiliations with commercial sites; or the user’s Twitter page consisting only of promotional tweets.
	Song lyrics/pop culture	Tweets that are lyrics from songs about menthol cigarettes or pop culture references. This could be hashtags with an artist’s name or popular television shows.
**Other tobacco or substances (tweets mentioning use of other tobacco or other substances)**		
	Marijuana	Mentions of marijuana (eg, loud, blunt, weed, spliff, mary jane, wax).
	Alcohol	Mentions of alcohol.
	Cigars/little cigars and cigarillos	Mentions of cigars, little cigars or cigarillos, including blunts or specific brands.
	Hookah	Mentions of hookah, waterpipe, shisha, or narghile for smoking tobacco or brands.
	Nicotine replacement therapy	Mentions of nicotine replacement therapy such as nicotine gum, patch, or lozenge, or specific brands.
	E-cigarette	Mentions of e-cigarettes, vaporizers, e-hookah, vape pens, etc, or of specific brands.
	Smokeless/snus	Mentions of smokeless tobacco or snus (eg, dip, chew, snuff, spit) or specific brands.
	Other tobacco or substances	Write-in mentions of other tobacco products (eg, pipe, roll-your-own) or brands, or other drugs (eg, LSD^a^, cocaine).
**Populations (groups associated with menthol cigarette use and menthol cigarette marketing)**		
	African Americans	African Americans or African American culture, image, or tradition or references to specific African American individuals in relation to menthol cigarettes.
	LGBT^b^	LGBT populations or LGBT culture, image, or tradition or references to specific LGBT individuals in relation to menthol cigarettes.
	Women	Women and menthol cigarettes or references to specific women and menthol cigarettes.
	Children/adolescents	Children and adolescents, minors, or underage smokers (<18 years in the United States) and menthol cigarettes.
	Other population	Write-in references to populations of people, does not include job categories (eg, rappers).
**Sentiment (attitudes toward menthol cigarettes)**		
	Positive	Positive sentiment about menthol cigarettes.
	Negative	Negative sentiment about menthol cigarettes.
	Neutral	Either no sentiment about menthol cigarette or mixed sentiments (both positive and negative) about menthol cigarettes.

^a^LSD: lysergic acid diethylamide.

^b^LGBT: lesbian, gay, bisexual, or transgender.

We used the codebook to code a random sample of 7000 tweets weighted by prevalence of keywords in the menthol cigarette-relevant dataset. Thus, even tweets with low-prevalence keywords had a probability of being selected equal to their prevalence in the larger dataset. Retweets were coded the same as the original tweet. We coded the entire tweet content of modified retweets, but coded sentiment on the modification alone. Each tweet was coded with as many categories as were applicable.

Next, 4 coders independently coded 500 tweets, reconciling after each 100 and clarifying the codebook, as needed, including adding or broadening codes. Coders also used “other” (write-in) responses to code for new categories that were not in the initial codebook. We added prevalent other categories to the codebook, and reviewed previously coded tweets for the presence of any applicable other categories. Once coders reached kappa of .8 reliability across raters and categories, individual coders coded the remaining tweets independently. Coders flagged unclear tweets, which were adjudicated by a second coder (SWR, CLJ, or MB). We excluded non-English language tweets and those that contained relevant keywords but were not about menthol cigarettes (eg, smoking on Newport Beach, or enjoying Newport, RI).

#### Themes

We identified potential themes and the topical content of the tweet related to perceptions of menthol cigarettes, based on prior research [18-21,43], and refined these themes during preliminary coding. For example, after preliminary coding, we added categories such as smoking behavior and tobacco industry marketing. We also broadened some categories that were not easily distinguishable in the open-ended tweet format compared with a closed-ended survey, for instance, collapsing medicinal effect and harm perceptions into a broader health category. Additionally, we derived one potential theme, African American image [18]) from research on a single subpopulation. We lacked information on user race/ethnicity, so we could not readily code information on users’ perception of menthol cigarettes and their cultural identity. Instead, to capture this concept, we coded references to several populations that may be associated with menthol cigarette use in a set of population codes (described below). These codes included the concept of image. Our final list of categories comprised taste or sensation, health, smoking behavior, cessation, addiction, tobacco control, tobacco industry and marketing, and other (low-prevalence categories written in by coders).

#### Populations

We coded menthol-related tweets if they specifically mentioned menthol cigarettes in relation to populations targeted by menthol marketing [9] and with a higher prevalence of menthol cigarette use [4,5]. Such populations included African Americans, LGBT populations, women, and children and adolescents. We also included an “other” category to capture other populations mentioned (eg, Latinos). Other groups were at low prevalence in the sample.

#### Other Tobacco or Substances

We coded tweets based on co-mentions of menthol cigarettes and alcohol, marijuana, other substances, or other tobacco products, including cigars, little cigars, cigarillos (including blunts), e-cigarettes, smokeless tobacco, and hookah. Perceptions of other tobacco and substances may interact with perceptions of menthol cigarettes and be associated with increased risk behaviors [44,45].

#### Tweet Characteristics

We coded 2 additional characteristics of tweets, including commercial versus noncommercial tweets based on a definition in a prior study (eg, branded promotional messages) [32]. Finally, we coded for whether the tweet included a song lyric or popular culture quotation (eg, a television show reference), a category that emerged from the data. We created lists of song lyrics with menthol cigarette references (eg, “Smoking mad Newports ’cause I’m due in court / For an assault that I caught in Bridgeport, New York”, from Everyday Struggle by The Notorious B.I.G.) and added them to the list as we identified new lyrics. After compiling a final list, we reviewed all coded tweets for known song lyrics.

#### User Smoking Status

We coded tweets for likely user smoking status (nonsmoker, former smoker, smoker, or unknown) because differences in perceptions of menthol cigarettes have been associated with smoking status in prior research [21]. Smokers were characterized by tweets discussing current cigarette smoking. Former smokers were characterized by tweets about past smoking or having quit smoking. Nonsmokers noted that they did not smoke or were opposed to smoking; antitobacco organization tweets were included. Users not mentioning their own smoking status in the tweet were coded as unknown.

#### Sentiment

We coded each tweet for sentiment toward menthol cigarettes. Sentiment categories were positive, negative, and neutral/unclear. If a tweet included both positive and negative sentiments (eg, wanting to quit menthols, but liking the taste), it was coded as neutral/unclear. Tweets mentioning actual smoking, or wanting or craving a menthol cigarette, without further comment were coded as positive toward menthol cigarettes.

### Analysis

Of the 7000 coded tweets, we excluded 343 (4.90%) as being either non-English or irrelevant to cigarette smoking or menthol cigarettes, with 95.1% retrieval precision and 90.4% retrieval recall within the HMC archive [46], leaving an analytic sample of 6657 tweets.

For these tweets, we provide univariate statistics of the frequency of tweets and provide qualitative narrative about frequent or notable content in each category. When examining differences in sentiment by other categories, we conducted chi-square tests to assess statistically significant differences in positive or negative sentiment toward menthol cigarettes.

## Results

### Frequency Analysis

Table 3 shows the frequency of themes, populations, other tobacco or substances, tweet characteristics, user smoking status, and sentiment.

**Table 3:** Frequency of menthol cigarette-relevant tweets by category (n=6657).

Category		n	%
**Themes**			
	Smoking	3983	59.83
	Taste/sensation	1676	25.18
	Addiction	1097	16.48
	Cessation	584	8.77
	Health	571	8.58
	Tobacco industry/marketing	390	5.86
	Other theme	299	4.49
	Tobacco control	295	4.43
**Population**			
	African American	745	11.19
	Women	587	8.82
	Child/adolescents	131	1.97
	Other population	28	0.42
	Lesbian, gay, bisexual, or transgender	22	0.33
**Other tobacco or substances**			
	Marijuana	1104	16.58
	Alcohol	221	3.32
	Cigar/little cigars and cigarillos	218	3.27
	Other substance	59	0.89
	E-cigarette	38	0.57
	Hookah	10	0.15
	Smokeless	3	0.05
**Tweet characteristics**			
	Song lyric/pop culture	1118	16.79
	Commercial	91	1.37
**User smoking status**			
	Smoker	4038	60.66
	Unknown	2141	32.16
	Nonsmoker	351	5.27
	Former smoker	127	1.91
**Sentiment**			
	Positive	3194	47.98
	Negative	2680	40.26
	Neutral/unclear	783	11.76

#### Themes

The majority of tweets about menthol cigarettes referenced smoking, taste, or sensation (eg, smell, coolness). About 17% referenced addiction (1097/6657), including explicitly discussing addiction, craving, or addiction-related behaviors such as chain smoking. All other themes—including cessation, health, tobacco industry and marketing, and tobacco control—were represented in fewer than 10% of the tweets coded. Tobacco control tweets (295/6657, 4.43%) included discussion of media campaigns or policy options including tax or price, minors’ access, smoke-free air laws, sales or marketing restrictions, or warning labels, or were tweets by tobacco control organizations. Fewer than 0.60% of tweets (40/6657) referenced a menthol cigarette ban; most were about potential EU action. Only 0.20% (13/6657) were by tobacco control organizations.

#### Populations

About 11% of tweets (745/6657) linked African Americans and menthol cigarette use. For African Americans, use of menthol cigarettes, especially Newport cigarettes, was viewed as linked with African American culture. This was particularly true for African American males. This linkage was viewed both positively (“@___: If you black and smoke cigarettes at least smoke Newports”) and negatively (“Why do black people have a fetish with Newport cigarettes?”). Stereotypical jokes or hashtags were also associated with this category (“#YouKnowYouBlack,” “#WaysToPissOffaBlackPerson”). Women were linked with menthol cigarettes in about 9% of tweets (587/6657). Linkage with menthol cigarettes was associated with negative attitudes toward women, such as the unattractiveness of women who smoke (“Hate when I see pretty girls smoking Newports, it really just ruins it”) or lack of class (“#ShesARatchetIf she smokes newports”). Few tweets referenced children and adolescents or LGBT populations.

#### Other Tobacco or Substances

Over 16% of tweets (1104/6657) referenced marijuana and menthol cigarettes. These tweets referenced co-use of marijuana and menthol, such as smoking a menthol cigarette to enhance the high of smoking marijuana, comparing excessive use of marijuana as similar to chain smoking menthol cigarettes, or assessing the relative harms or appeal of marijuana and menthol cigarettes. These tweets usually judged marijuana as less harmful and more appealing than menthol cigarettes. Fewer tweets (218/6657, 3.27%) focused on dual use of menthol cigarettes and cigar products such as Black and Mild or Swisher Sweets, or blunts used for smoking marijuana. Alcohol and menthol cigarettes were mentioned in 3.32% (221/6657) of the tweets and discussed smoking behavior and drinking. Other tobacco products were rarely mentioned in conjunction with menthol cigarettes.

#### Tweet Characteristics

Only 1.37% of tweets (91/6657) in the sample were commercial tweets, such as tweets offering links to coupons or discounts. Just under 17% of tweets (1118/6657) referencing menthol cigarettes were song lyrics or popular culture quotations (such as a game show sketch from *Chappelle’s Show*, “I Know Black People,” famously asking the question “Why do black people like menthol cigarettes?”). We identified 30 songs or quotations tweeted in the sample.

#### User Smoking Status

The majority of tweets were likely by smokers (4038/6657, 60.66%) or users with unknown status (2141/6657, 32.16%).

#### Sentiment

Sentiment toward menthol cigarettes in the tweets was mixed. Overall, 47.98% (3194/6657) expressed positive sentiment toward menthol cigarettes and 40.26% (2680/6657) of tweets expressed negative sentiment. The remainder were neutral or unclear.

### Sentiment Analysis

**Figure 2:**
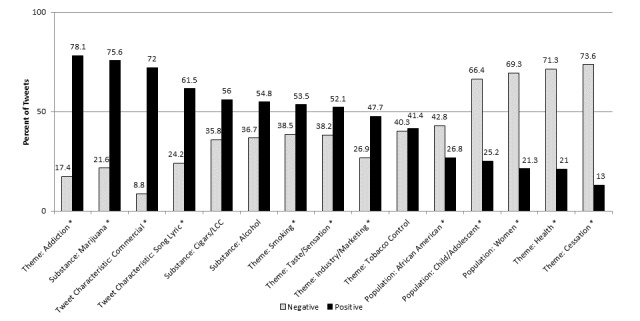
Positive and negative sentiment toward menthol cigarettes by domains and categories (n=6657). LLC: little cigars and cigarillos. *Pearson chi-square comparison between positive and negative sentiment, significant at *P*<.05.

Sentiment varied by category across themes, populations, other tobacco and substances, and tweet characteristics (omitting categories with <1% prevalence) Figure 2 shows. Based on a chi-square test, tweets coded as referencing themes of addiction (including concepts such as craving or desire for a menthol cigarette), smoking, taste or sensation, and industry or marketing; marijuana as an other substance; and tweet characteristics of commercial tweets and song lyrics were significantly more likely to be positive than negative toward menthol cigarettes. However, both smoking and taste or sensation themes had close to 40% negative sentiment. Positive smoking and taste sentiment included positive attributes of smoking (relaxing, relieving stress, taking smoke breaks) and enjoyment of menthol (pleasant minty taste, soothing or cooling sensation). Negative sentiment included negative mood (stress, anxiety, “bad nerves”), negative feelings about other people smoking (“#ItsNotCuteWhen you smoking ports <<<<”), or dislike of the taste or smell of menthol cigarettes (“Smoking a newport. #gross,” “I hate the scent of newports!! Cigarettes in general Smell terrible, but especially newports!”).

Tweets referencing themes of cessation and health, and populations of women, children and adolescents, and African Americans were significantly more likely to be negative toward menthol cigarettes. Almost no cessation-related tweets noted proven quit methods such as the use of FDA-approved cessation aids, talking with a physician, or using a quitline [47]. Many noted plans to quit after smoking a last cigarette or pack, or asked for support for their quit attempt (“Today is my last day smoking cigarettes....waving good bye Newports! Wish me luck!!”). A few noted trying to quit by using an e-cigarette (14/6657 tweets). A small number referenced making a quit attempt in relation to the US Centers for Disease Control and Prevention’s Tips from Former Smokers campaign (“Seriously those commercials of the people with the hole in their throat made me quit smoking I haven’t had a newport in days”) [48]. Some also tried to quit by switching to a nonmenthol cigarette (“I need to get off menthols. I need to quit but if I start smoking something disgusting it may be easier.”).

Most health tweets were negative, noting negative health consequences of smoking. However, positive tweets related to health expressed a misperception of menthol cigarettes as having medicinal use when sick (“Menthol cigarettes are my saviour with this cold”). We rarely found harm perceptions of menthol as less harmful than other cigarettes (“Oh natural menthol healthy cigarettes”), but the converse message that menthol was harmful was more prominent (“SMOKERS (menthol or nonmenthol) really WILL HAVE stroke. So better stop smoking.” “Menthol cigarettes? So they soothe your throat while giving you cancer? Kinda like being killed by a clown.”). Regarding populations, negative sentiment toward menthol cigarettes was driven by negative views of smoking among children and adolescents, women, and African American populations. Tweets referencing cigars, alcohol, and tobacco control had no significant differences between positive and negative sentiment toward menthol cigarettes.

**Figure 3:**
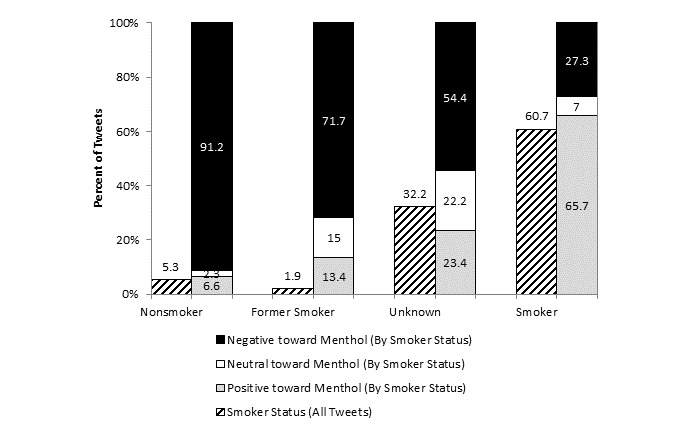
Tweets by smoker status and sentiment (n=6657).

Among smokers, 65.70% (2653/4038) expressed positive sentiment toward menthol (“Listening to music and smoking a port so relaxed”) (Figure 3). Only 27.34% of smokers (1104/4038) reported negative sentiment toward cigarettes in general (“I cant be doin these menthols man.! Gotta Quit Smoking period. Maybe tomorrow”) or specifically toward menthol-type cigarettes or a menthol cigarette brand (“Had a mayfair menthol, its like smoking mouthwash”). Smoker conversations about menthol cigarettes were dominated by discussion of smoking behavior, addiction or craving, taste preference, and marijuana.

Nonsmokers (320/351, 91.2%) held predominately negative sentiments about menthol cigarettes and smoking in general (“All the girls at my job be smoking them Newports and had the nerve to offer me one. No ma’am, never.”). Former smokers were generally negative about menthol (91/127, 71.7%) (“I remember when I used to be addicted to Newport cigs now the smell of cigarette smoke gives me a headache #happyiquit #cigsarenasty”). However, almost 30% (36/127, 28.4%) of former smokers were positive or ambivalent about menthol (“even though i quit smoking im tempted to try some marlboro menthol lights, i heard they stink less and are less harmful”).

## Discussion

### Principal Findings

Tweets about menthol cigarettes were most frequently about themes of smoking, taste or sensation, and addiction; African American populations; marijuana as an other substance; or song lyrics or pop culture references. Menthol content on Twitter was also driven by smokers who were more positive than others about menthol cigarettes. A prior study of tobacco-relevant content on Twitter found that most tweets were about personal experiences of use [34]. Liking or disliking the taste or sensation of menthol cigarettes found in a quarter of the tweets and in prior research [18,23] are understandable reasons for smoking menthol rather than nonmenthol cigarettes.

Additionally, Twitter users, particularly smokers, frequently mentioned marijuana and menthol cigarettes, and such tweets tended to be positive. This type of linkage has been found related to use of these products, with adolescent menthol smokers more likely to use marijuana in the past 30 days than nonmenthol smokers [49,50]. Similar to the effect of Twitter content on perceptions of the use of hookah [37] and marijuana [51], these positive views of menthol could normalize menthol cigarette use among the Twitter followers of these users.

Discussion over Twitter focused on smokers’ concerns and tended to dwarf public health concerns, such as smoking cessation, health, and tobacco control. Some conversations about menthol cigarettes were cessation related but, as with other Twitter conversations about cessation, they lacked discussion of evidence-based strategies [52]. As in prior work, a small number of menthol smokers on Twitter may have perceived that switching to a nonmenthol cigarette is a good way to quit [26]. As in prior studies [18,53,54], we found that some smokers saw a medicinal effect of menthol cigarettes when they were sick. Studies have found that African American smokers may perceive health benefits of menthol or view menthol cigarettes as less harmful than nonmenthol cigarettes—beliefs that may interfere with quitting [14,18,20,23,24,26]. However, this view of menthol cigarettes as less harmful was not prevalent on Twitter and may reflect differences in broader population views of menthol. For instance, a national study of US smokers and nonsmokers found that 45.8% of adults believed that menthol and nonmenthol cigarettes were equally harmful; however, a sizable minority did not know whether menthol cigarettes were more or less harmful than nonmenthol cigarettes (40.8%) [21]. Few conversations on Twitter reflected concern for tobacco control policies. There was limited discussion about a menthol cigarette ban in the EU. After the TPSAC report in 2011 [12], but before an advanced notice of proposed rulemaking on menthol in cigarettes in 2013 [55], there was almost no Twitter discussion about a possible menthol cigarette ban in the United States or any other jurisdiction.

Finally, tweets reflected linkage of menthol cigarettes with African American populations historically targeted by menthol marketing and that have higher use prevalence [4,56]. Prior work has identified this “African Americanization” through targeted marketing and financial support for African American organizations [56]. African American menthol smokers have recognized that they have been targeted by menthol cigarette advertising [26]. This linkage between menthol cigarettes and African Americans is also seen on Twitter and is characterized by negative sentiment toward menthol cigarettes. Unfortunately, this negative sentiment seems to be driven by negative stereotypes about African American smokers rather than by rejection of the targeted marketing of menthol cigarettes to African Americans.

### Strengths and Limitations

The strengths of this study are that it included a full year of global Twitter data from the Firehose, representing the entire corpus, rather than a sampling of Twitter content. Limitations are that, because we drew menthol content only from existing smoking and cigarette datasets, we may have missed menthol cigarette-relevant Twitter content without those terms. Additionally, due to this limitation, our research cannot be generalized to all tweets. We could not use Kool and Salem brand names as keywords because they were not precise enough to characterize menthol cigarette-relevant conversations. Future research in this area should consider using the menthol cigarette keywords beyond these existing archives and also examine potential substitution of menthol cigarettes with menthol flavors of other tobacco products such as e-cigarettes or cigars. The study lacked information about demographic or geographic information of Twitter users to understand differences in perceptions. However, themes found in prior research about menthol cigarettes [18-21,43] were also found in this study, suggesting these perceptions may be shared broadly. Future research can focus more in-depth on discussion of menthol cigarettes related to different populations. Another limitation was the cross-sectional nature of our analysis. Though the analysis was appropriate given our interest in the overall frequency of content about menthol cigarettes in this time period, a longitudinal analysis could provide information about changes in perceptions of menthol cigarette Twitter content.

### Conclusion

Our findings present a relatively new way of assessing public opinions of menthol cigarettes through Twitter messages. Most messages are generated by smokers who have more positive sentiment toward these harmful combustible products. Misperceptions of menthol cigarettes having medicinal effects are prevalent, and positive linkage with marijuana is common. Addressing these common misconceptions and denormalizing menthol cigarette use, particularly for African American smokers, could ultimately save lives. In the United States, a menthol cigarette ban would be estimated to avert over 600,000 deaths by 2050, almost one-third among African Americans [57]. Framing public communication about menthol cigarettes to denormalize use in both online and offline forums is especially critical in light of potential regulation by the FDA, the EU, other jurisdictions, and localities. Future analyses could also use Twitter to examine reaction to regulations restricting menthol cigarettes among likely smokers and nonsmokers.

## Appendix

Multimedia Appendix 1 Detailed study codebook.

## Abbreviations

EU: European Union
FDA: Food and Drug Administration
HMC: Health Media Collaboratory
LGBT: lesbian, gay, bisexual, or transgender
TPSAC: Tobacco Products Science Advisory Committee
