# Supplementary material for: Perceptions of Menthol Cigarettes Among Twitter Users: Content and Sentiment Analysis
Source: J Med Internet Res. 2017 Feb 27;19(2):e56. doi: 10.2196/jmir.5694 (PMC5348619; doi:10.2196/jmir.5694)
Supplement: Multimedia Appendix 1 [file jmir_v19i2e56_app1.pdf]

Codebook

Section 1. Tweet Information

Tweet Text: The text of the tweet to code is here. Only code based on tweet content including hashtags, emoticon, or emojis, but not on links or user names included in the tweet. For retweets or song lyrics, code based on tweet content. For modified tweets or modified song lyrics, code theme based on entire content, but code sentiment based on tweet user comment only (e.g., “Smoking Cigs are disgusting RT @user Newports are good!” (you would code as sentiment negative)). If the tweet text is garbled or has special characters due to conversion then enter the mlink into a browser to see the tweet as formatted on twitter – code text. (e.g., > means greater than >)

Mlink: tweet link – can enter this into a web browser to see the tweet with special characters or emojis. If the account is no longer active than the link may not work.

mID: unique identifier for Tweet

Section 2: Inclusion/Exclusion

Inclusion/Exclusion criteria for determining if a tweet was related to menthol cigarettes

**Inclusion Criteria**	**Has to do with menthol cigarettes or smoking menthol cigarettes – also include implicit comparisons to menthol cigarettes (e.g., “Someone bring me a non-menthol cigarette;” “Non-menthol cigarettes are gross”). This also includes references to predominately menthol brands of cigarettes (e.g., Newport, Kool, and Salem) unless non-menthol versions of these brands (e.g., Newport Red) are explicitly the focus of the tweet. Tweets specifying dual use of marijuana and menthol cigarettes should be included.**
**Exclusion Criteria**	**IF ANY Exclusion criteria is Yes, then do not code further**
**Non-menthol Relevant**	Exclude things that are not related to menthol cigarettes, including tweets only related to menthol electronic cigarettes, smoking or cigarettes in general, tobacco products other than cigarettes, marijuana, and references to menthol that are part of a user name unless the tweet includes relevant menthol cigarette content.
**Non-Smoking relevant**	Exclude tweets not related to smoking or tobacco products (including e-cigarettes) at all (e.g., “smoking hot girl at Newport Beach”)
**Non-English Tweet**	Exclude any tweet that is not in English. If the tweet is partial in English and partially in another language, exclude.

Section 3. Tweet Themes

Taste Preference/Sensation

Inclusion = YES: Tweets referencing the taste, smell, or sensation (e.g., cooling, minty, refreshing) of menthol cigarettes either in relation to non-menthol cigarettes or in general. Tweets can be either positive or negative about taste preference or sensation (e.g., liking or disliking menthol cigarettes; preferring a weaker or stronger menthol flavor). Tweets can refer to the user's own cigarette use, that of others, or menthol cigarette use in general. General tweets about preference or lack of preference for menthol cigarettes, such as "I like menthol cigarettes" should be included. Tweets that fit the criteria for menthol cigarette relevance, but where the statement about taste/sensation is related to cigarettes in general should also be included in this code (e.g., "Cigarettes taste good. #Menthol"). Included brand preference between menthol brands (e.g., Newport better than Salem).

Exclusion = NO: Tweets that do not reference taste, smell, or sensation of using menthol cigarettes should be excluded. Tweets that are only about craving for menthol cigarettes (e.g., "I want a menthol cigarette" or about health effects of menthol cigarettes (e.g., "menthol cigarettes make me cough") should be excluded.

Health Concerns

Inclusion = YES: Tweets about perceived health benefits of menthol (vs. non-menthol cigarette use or other product use) or ideas about the harms/risks of menthol cigarette (e.g., they are more or less harmful than non-menthol cigarettes or other products). This code should also include beliefs about medicinal effects of menthol cigarettes (e.g., using or not using such cigarettes if the user is sick, or if such cigarettes make someone sick). Ideas about positive and negative health concerns about menthol cigarettes should be included or the effects of menthol cigarettes on the user or another (e.g., his voice sounds like he's been smoking Newport). In a tweet that meets the menthol inclusion criteria, general statements about health concerns of cigarettes in general should be included (e.g., "Cigarettes make me cough. #menthol") .

Exclusion = NO: Tweets that do not reference health concerns or harms of menthol cigarettes should be excluded. Any tweet that fits an exclusion for menthol relevance (see definition above) should also be excluded.

Cessation

Inclusion = YES: Tweets about menthol cigarettes and cessation. These may include the desire or lack of desire to quit smoking menthol cigarettes; beliefs about whether menthol cigarettes are harder/easier to quit than non-menthol cigarettes, or discussion of quitting strategies (for instance switching to or from menthol to help with a quit attempt). Also include if the person used to smoke menthol cigarettes, but has now quit. Tweets can either reference the user or another person (e.g., urging someone else to quit or not quit).

Exclusion = NO: Tweets that are not about cessation regarding menthol cigarettes. Any tweet that fits an exclusion for menthol relevance (see definition above) should also be excluded.

Addiction
Inclusion = YES: Tweets about menthol cigarettes and addiction. These may include addiction or lack of addiction to menthol cigarettes; or beliefs that menthol cigarettes are more/less addictive than non-menthol cigarettes. Tweets can include cravings or lack of craving (urges to smoke) for menthol cigarettes, or desire or lack of desire for a menthol cigarette ("I really need to smoke a cigarette now #addicted"). References to chain smoking should be included. Tweets can either reference the user or another person.
Exclusion = NO: Tweets that are not about addiction regarding menthol cigarettes. Any tweet that fits an exclusion for menthol relevance (see definition above) should also be excluded.

Smoking behavior
Inclusion = YES: Tweets about the act or process of smoking menthol cigarettes including time of day of smoking (e.g., morning, evening), place of smoking (e.g., inside, outside), or social context of smoking (e.g., alone, with friends, while drinking or smoking marijuana). Can include smoking behavior by the user or others (e.g., My mom was just smoking in the car)
Exclusion = NO: Tweets that are not about smoking menthol cigarettes. Any tweet that fits an exclusion for menthol relevance (see definition above) should also be excluded. If tweets are only about desire or craving for a menthol cigarettes and not about actually smoking (I really want a Newport now) code as addiction instead.

Tobacco Control Policies
Inclusion = YES: Tweets regarding menthol cigarettes and tobacco control polies or the impact of tobacco control policies. These may including the impact of price (e.g., menthol cigarettes are $10 a pack?!), smokefree air laws (e.g., I can't smoke inside now), references to the potential for a menthol ban, anti-tobacco media campaigns, or sales and marketing restrictions on menthol cigarettes. Tweets by anti-tobacco advocacy organizations about menthol should also be included.
Exclusion = NO: Tweets that do not reference menthol cigarettes and tobacco control policies or their impact should be excluded. Any tweet that fits an exclusion for menthol relevance (see definition above) should also be excluded.

Tobacco Control Policy Topic – Only code if Tobacco Control Policy is Yes

- Tax or Price – Cost of menthol cigarettes, price of menthol cigarette pack or container, or about tobacco taxes from legal sources. Also include illicit sale prices/costs (e.g., got $5 packs here).
- Smokefree air – References to where people are allowed to smoke or not smoke due governmental or institutional regulations or policies (also include personal policies like home or car bans), specific mention of smokefree or clean air policies. Taking a "smoke break" is not by itself a reason to use this code.
- Menthol Ban – References to a ban on menthol cigarettes or concerns about the possibility of a ban.
- Media campaigns – References to anti-smoking or anti-tobacco media campaigns (e.g., tips from a former smoker, real cost, truth)

- Anti-tobacco organizations/advocate – references to particular anti-tobacco organizations or tweets by those organizations as @users or rt from those users. Only code anti-tobacco org/advocate if the tweet content does not have to do with other policy topics.
- Sales or Marketing restrictions – references to advertising or labeling or other point of sale restrictions including graphic warning labels, bans on self-service of tobacco products, sale of loosies, restrictions on promotions like gifts with purchase, banned flavored cigarettes (NOT other flavored tobacco products).
- Minor's access restrictions – references to youth sales restrictions or minor's access policies, include mentions of people giving tobacco products to minors as well. Note: make sure to only use this code to refer to minors not just a son or daughter who may be an adult.
- Other policy – Any other policy topic – if this is selected write in what other policy in the other theme mention box as OTHER POLICY: fill in policy type.

Industry/Marketing

- Inclusion = YES: Tweets regarding the advertising or labeling or packaging of menthol cigarettes. These may including reference to tobacco ads, how the pack or logo looks, include ads or labels about particular brands. Also include references to cheap/discount brands of cigarettes or selling cigarettes online (including commercial tweets) or cigarette coupons/promotions, or to new brands or types of cigarettes (e.g., slims, longs, etc). May also include references to tobacco companies or pro-tobacco organizations.
- Exclusion = NO: Tweets that do not reference menthol cigarette industry marketing, advertising or packaging should be excluded. Any tweet that fits an exclusion for menthol relevance (see definition above) should also be excluded.

Other Theme Mention – code additional themes not captured above.

Section 4. User Smoker Status

User Smoker Status. Code based on tweet content even if the user did not generate the original content. For retweets or song lyrics, code smoking status based on tweet content. For modified tweets or modified song lyrics, code smoking status based on modified content only.

- **Smoker.** User writing tweet is likely to be a smoker. (Note: if person is giving tobacco products to another than code as smoker)
- **Former Smoker.** User writing tweet is likely to be a former smoker
- **Non-smoker.** User writing tweet is likely to be a non-smoker**.** Also use this code for tweets from anti-tobacco organizations.
- **Unknown.** Cannot determine smoking status of user writing tweet.

Section 5. Tweet Characteristics

Commercial
Inclusion = YES: Commercial tweets are defined by the presence of any of the following: branded promotional messages; URLs linking to commercial websites; usernames indicating affiliations with commercial sites; or the user's twitter page consisting only of promotional tweets (i.e. spammer accounts). Examples of tweets to include are those promoting sales of menthol cigarettes or offering coupons for menthol cigarettes.
Exclusion = NO: “Organic” tweets are non-sponsored; they reflected individual opinions or experiences or linked to non-promotional content. Any tweet that fits an exclusion for menthol relevance (see definition above) should also be excluded.

Song Lyrics/Pop Culture
Inclusion = YES: Tweets that are lyrics from songs about menthol cigarettes or pop culture references. [See list for known lyrics/pop culture reference – repeated or similar tweets that are not retweets or tweets that rhyme are likely to be song lyrics]. May have hashtags with artist name or popular tv shows.
Exclusion = NO: Tweets that are not lyrics from popular songs about menthol cigarettes or pop culture references. Any tweet that fits an exclusion for menthol relevance (see definition above) should also be excluded. [note that a trending hashtag was #2ChainzLyrics or #Things2Chainzsays or similar – These are not actually lyrics by rapper 2Chainz, they are jokes and should be coded as no.]

Check drop down list for known lyrics/pop culture references. If you think you have a lyric or pop culture quotation– check on internet to confirm and then write in **correct** lyrics in SongLyricOther (not what is in the quote which may only be an approximation of the lyric]

Section 6. Other tobacco or substances

Other tobacco or substances. Only code for tweets that are menthol cigarette relevant

- **Marijuana.** Code yes if tweet mentions marijuana (e.g., loud, blunt, weed, spliff, mary jane, wax, etc.). [add additional slang to codebook]
- **Cigars/LLC.** Code yes if tweet mentions cigars, little cigars or cigarillos including blunts. Include mention of specific brands (e.g., Dutchmaster, Black and Mild, etc.)
- **Hookah.** Code yes if tweet mentions hookah, waterpipe, shisha, narghile, etc. for smoking tobacco etc. If hookah is used for marijuana code marijuana as well.
- **NRT.** Code yes if tweet mentions nicotine replacement therapy like nicotine gum, patch, lozenge (don’t include mentions of regular gum). Include mention of specific brands, Nicorette, Nicotrol, etc.)
- **Ecigarette.** Code yes, if tweet mentions e-cigarettes, vaporizers, e-hookah, vape pens, etc. [add additional slang to codebook] Include mention of specific brands, BLU, NJOY, etc.

- **Smokeless/Snus.** Code yes if tweet mentions smokeless tobacco or snus (e.g., dip, chew, snuff, spit) [add additional terms to codebook] or specific brands (e.g., camel snus, Redman etc.)
- **Other tobacco or substances:** Write-in if tweet mentions other tobacco products (e.g., kretek, bidis, pipe, roll-your-own RYO) or brands of those products or other drugs (e.g., LSD, cocaine)

Section 7. Populations

Populations are those that have more prevalent use of menthol cigarettes and have experienced targeted marketing of menthol cigarettes.

Note: All codes in this category should be coded based on the text of the tweet only. It should not include coding based on pictures or links included with the tweet.

African-Americans

Inclusion = YES: Tweets that reference menthol cigarettes in relation to African Americans, African-American culture, image, or tradition. Tweets should also be included that contain an implicit reference or comparison to African-Americans (e.g., White people don't smoke menthol cigarettes). Include references to specific African-American individuals in relation to menthol cigarettes (e.g., "Wiz Kalifa and Rick Ross…")

Exclusion = NO: Tweets that do not reference menthol cigarettes in relation to African Americans, African-American culture, image, or tradition. Any tweet that fits an exclusion for menthol relevance (see definition above) should also be excluded. Do not include just because of a reference to an African-American in the tweet if the reference is not also about the specific person in relation to menthol cigarettes (e.g., I was watching Oprah and smoking a Newport)

LGBT

Inclusion = YES: Tweets that reference menthol cigarettes in relation to Gay, Lesbian, Bisexual or Transgender people, LGBT culture, image, or tradition. Tweets should also be included that contain an implicit reference or comparison to LGBT (e.g., Straight people don't smoke menthol cigarettes).

Exclusion = NO: Tweets that do not reference menthol cigarettes in relation to LGBT people, culture, image, or tradition. Any tweet that fits an exclusion for menthol relevance (see definition above) should also be excluded. Do not include just because of a reference to an LGBT person in the tweet if the reference is not also about the specific person in relation to menthol cigarettes (e.g., I was watching Ellen and smoking a Newport)

Women

Inclusion = YES: Tweets that reference menthol cigarettes in relation to women. Tweets should also be included that contain an implicit reference or comparison to women (e.g., Men don't

smoke menthol cigarettes). Include references to specific women smoking menthol include, “my aunt, grandmother etc. as well as famous women.
Exclusion = NO: Tweets that do not reference menthol cigarettes in relation to women. Any tweet that fits an exclusion for menthol relevance (see definition above) should also be excluded. Do not include just because of a reference to a women in the tweet if the reference is not also about the specific person in relation to menthol cigarettes (e.g., I was watching Oprah and smoking a Newport)

Children/Youth
Inclusion = YES: Tweets that reference menthol cigarettes in relation to children/youth and minors who are underage smokers (less than 18 in US). This could include tweets about current youth (e.g., I saw a 13 year old smoking a menthol cigarette) or past youth (e.g., I started smoking menthol cigarettes when I was 14). Tweets should also be included that contain an implicit reference or comparison to youth (e.g., Adults don’t smoke menthol cigarettes)
Exclusion = NO: Tweets that do not reference menthol cigarettes in relation to children/youth/minors. Any tweet that fits an exclusion for menthol relevance (see definition above) should also be excluded. Do not include just because of a reference to youth/child in the tweet if the reference is not also about the specific person in relation to menthol cigarettes (e.g., After I smoke this Newport I need to go pick up my kid). Exclude tweets that reference a family relationship (e.g., son or daughter) with someone who may be an adult.

Other population Mention
Include this if population mention is yes, but is not a population listed above. Only use this to include references to an entire population of people, do not include job categories (e.g., rappers) or references to individual other types of people. Write-in response

Section 8. Sentiment

Sentiment – code this based on overall impression.

- Positive toward menthol: Tweet is generally positive about menthol cigarettes.
- Negative toward menthol: Tweet is generally negative about menthol cigarettes.
- No Sentiment: No sentiment expressed about menthol cigarettes identified, cannot determine whether the sentiment is negative or positive; or both positive and negative sentiments about menthol cigarettes are present.
